# Giant Central Ossifying Fibroma of the Anterior Maxilla and Maxillary Sinus With Orbital Floor Extension: A Case Report

**DOI:** 10.1002/ccr3.73803

**Published:** 2026-10-26

**Authors:** Ahmed Alashwal, Naibah Rajeh, Arham M. Alkherbash, Ahmed Madfa

**Affiliations:** ^1^ Department of Oral and Maxillofacial Surgery, Faculty of Dentistry Emirates International University Sana'a Yemen; ^2^ Department of Conservative Dentistry, Faculty of Dentistry Sana'a University Sana'a Yemen; ^3^ Department of Restorative Dental Science, College of Dentistry University of Ha'il Ha'il Saudi Arabia

**Keywords:** cemento‐ossifying fibroma, enucleation, fibro‐osseous lesion, maxillary sinus, orbital floor, ossifying fibroma

## Abstract

A 25‐year‐old woman presented with a giant conventional ossifying fibroma of the anterior maxilla extending into the maxillary sinus and elevating the orbital floor. Complete intraoral enucleation preserved the lateral incisor. Histopathology confirmed conventional ossifying fibroma. No recurrence occurred at 12 months, supporting conservative treatment in carefully selected, well‐circumscribed lesions.

## Key Clinical Message

Giant conventional ossifying fibromas of the maxilla may be managed conservatively when the lesion is well circumscribed, encapsulated, and separable from vital structures. Careful patient selection, complete enucleation, and long‐term clinical and radiological follow‐up are essential.

## 1 Introduction

Benign fibro‐osseous lesions of the craniofacial skeleton comprise a heterogeneous group of disorders characterized by replacement of normal bone with cellular fibrous tissue containing variable amounts of mineralized material, including woven and lamellar bone and cementum‐like deposits [1, 2, 3]. The three main conditions seen in clinical practice are fibrous dysplasia, cemento‐osseous dysplasia, and ossifying fibroma. These lesions have significant histological similarities, but their pathophysiology, biological behavior, and therapy are essentially different. As a result, a reliable diagnosis necessitates the combination of clinical, radiographic, and microscopic observations rather than relying just on histology [3, 4]. Making the right differentiation is crucial since a real tumor may require total excision, while a developing dysplastic lesion may only require observation or contouring.

Within this spectrum, central ossifying fibroma (COF) is considered a benign but potentially expansile tumor. The term “cemento‐ossifying fibroma” was reinstated in the fourth edition of the World Health Organization's (WHO) Classification for lesions that occur in the jaws' tooth‐bearing regions because of their suggested connection to the periodontal ligament and its multipotent mesenchymal cells, which are capable of producing bone, cementum, and fibrous tissue [1]. Juvenile trabecular and juvenile psammomatoid ossifying fibromas were categorized as separate fibro‐osseous tumors in the fifth edition, which significantly improved the diagnostic framework [2]. The exact odontogenic versus osseous origin of COF is still up for debate [5, 6, 7], but its clinical significance is evident: unlike fibrous dysplasia, which typically stabilizes during skeletal maturation, COF demonstrates autonomous neoplastic progression and, as a result, usually needs to be completely removed [3, 8]. With documented female‐to‐male ratios of roughly 2:1 to 5:1, COF exhibits a clear female inclination and is most common in the second to fourth decades of life [9, 10, 11]. The most commonly impacted area is the mandible. Chang et al. reported mandibular involvement in 93% of 28 cases, while Titinchi and Morkel documented posterior mandibular involvement in 55.5% of 61 patients [10, 11]. On the other hand, lesions developing in the front maxilla are especially uncommon, and maxillary involvement is far less prevalent [9]. Extension into the sinonasal tract and maxillary sinus is even less common; only a few number of cases have been reported by multicenter and specialized rhinological studies [12, 13, 14].

The anatomical characteristics of the maxilla may allow COF to achieve substantial dimensions before becoming clinically evident. The pneumatized maxillary sinus provides a relatively low‐resistance space into which an expanding lesion can extend, potentially resulting in delayed presentation with facial asymmetry, nasal obstruction, dental displacement, or orbital involvement [13, 15]. Radiographically, COF is typically well circumscribed and may demonstrate progressive mineralization from predominantly radiolucent to mixed and ultimately radiopaque appearances [8, 16]. CT is particularly important for defining osseous expansion, sinus occupation, tooth displacement, and orbital relationships, whereas MRI may be required to evaluate soft‐tissue, orbital, or intracranial extension [15, 17].

Surgical excision remains the definitive treatment, although the optimal extent of surgery remains controversial. Conservative procedures, including enucleation and curettage, are commonly used for well‐demarcated lesions, whereas block or segmental resection is generally reserved for extensive, recurrent, or aggressive lesions [11, 18, 19]. Reported recurrence rates vary substantially, from 6.7% in a large series managed predominantly by curettage to 12% in a systematic review, and up to 23% in maxillary and sinonasal lesions and 28.6% in pediatric jaw lesions [8, 11, 12, 20]. This variability reflects differences in tumor subtype, anatomical location, surgical technique, completeness of excision, and follow‐up duration.

Evidence on conservative management of giant anterior maxillary COFs with sinonasal and orbital extension remains limited. We report a giant COF successfully treated by complete conservative enucleation with preservation of the adjacent dentition. This case reports the clinical, radiological, and histopathological features that supported this approach in selected lesions with distinct capsular demarcation, rather than proposing a general treatment strategy.

## 2 Case History/Examination

### 2.1 Clinical Presentation and Examination

A 25‐year‐old woman arrived at the Oral and Maxillofacial Surgery Clinic with a painless, gradually increasing edema of her left midface. The lesion had been observed about five years prior and had progressively become larger. Pain, paresthesia, discharge, epistaxis, epiphora, diplopia, and visual impairment were all denied by her. There was no history of radiation therapy, prior surgery, or face trauma. Her familial and medical histories were ordinary; she had no history of numerous fibro‐osseous lesions, hyperparathyroidism, or jaw tumors. Serum calcium, phosphate, alkaline phosphatase, and parathyroid hormone were all within reference ranges in routine hematological and biochemical tests, ruling out an underlying metabolic issue and offering no indication of hyperparathyroidism–jaw tumor syndrome.

Extraoral examination revealed significant left facial asymmetry, including elevation of the upper lip, effacement of the nasolabial fold, and a solid, non‐tender midfacial edema. There was no lymphadenopathy and the skin above was normal. Vision, extraocular motions, and infraorbital feeling were all unaffected by proptosis or globe dystopia, according to an ocular examination (Figure 1). The left anterior maxilla was affected intraorally by a bony‐hard, non‐tender swelling that resulted in labial and palatal enlargement as well as vestibule obliteration. The central incisor displayed Grade II mobility, and both the lateral and central incisors were displaced. There was no indication of an odontogenic infection, and the remaining teeth were vital (Figure 2).

**FIGURE 1:**
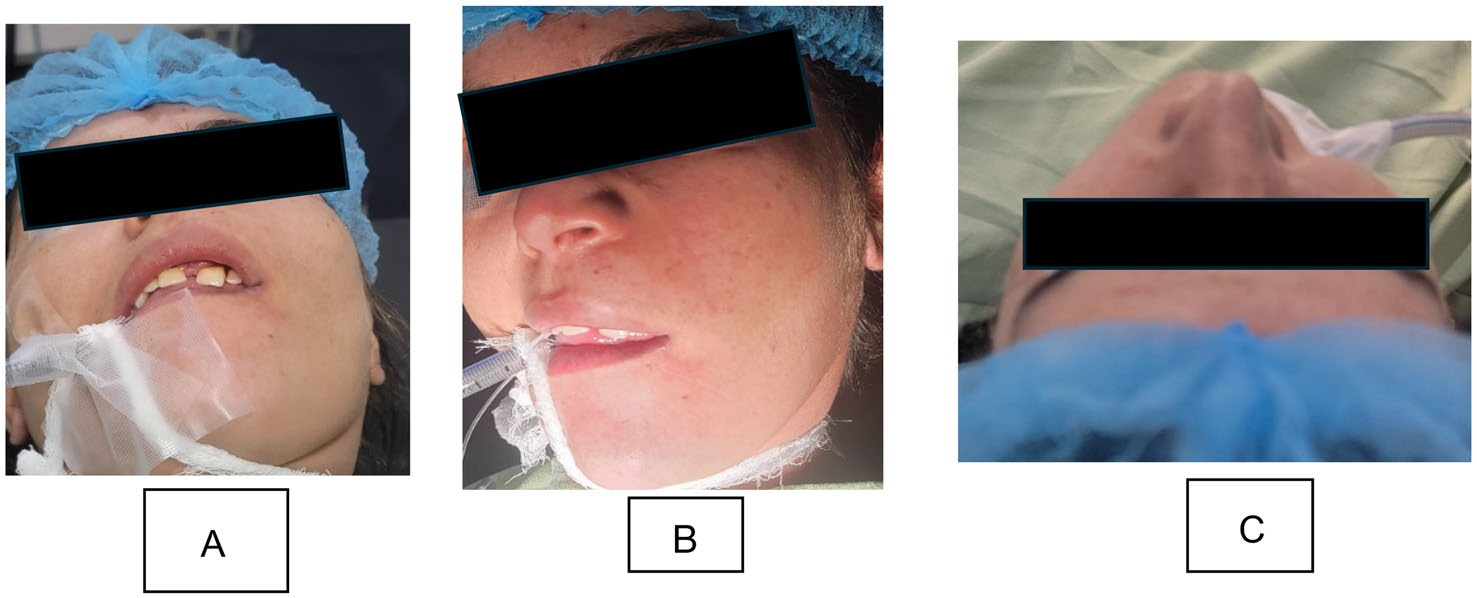
Pretreatment extraoral photographs showing facial asymmetry on the left side with effacement of the nasolabial fold and elevation of the upper lip: (A) frontal view; (B) lateral view; (C) worm's‐eye view.

**FIGURE 2:**
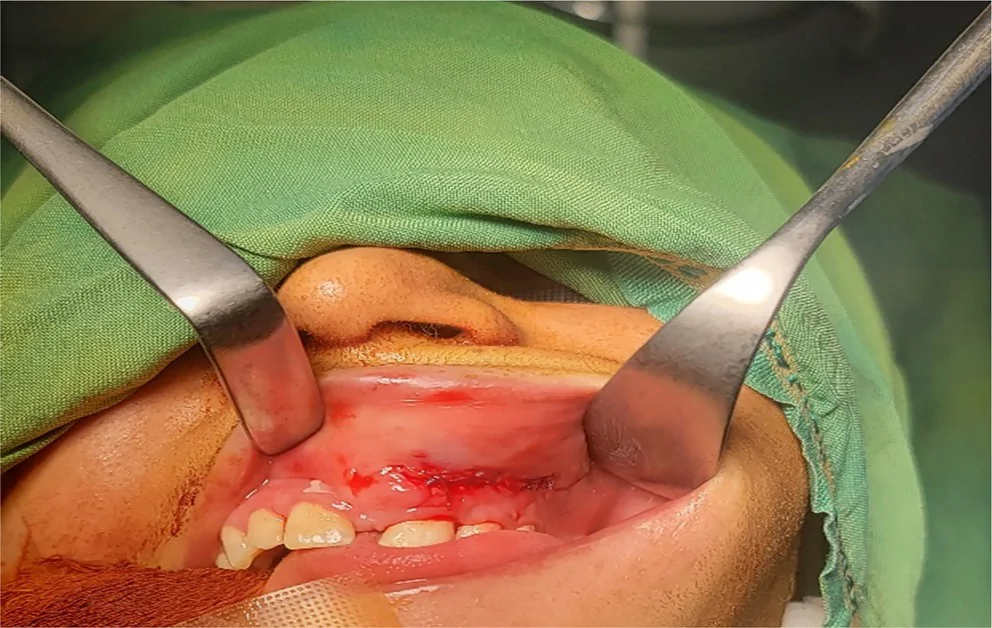
Pretreatment intraoral photographs showing bony‐hard expansion of the left anterior maxillary alveolus with labial and palatal expansion and normal overlying mucosa.

### 2.2 Radiological Investigations

A distinct, expansile lesion centered on the left anterior maxillary alveolus and spreading superiorly into the maxillary sinus to the level of the orbital floor was visible on computed tomography (CT). The lesion had a heterogeneous internal density, with mixed hypodense and hyperdense components that were compatible with increasing mineralization. Its dimensions were roughly 7 × 6 × 2 cm. The maxillary antrum's anterior, lateral, and posterior walls were noticeably thinned and expanded, but there was no obvious cortical damage or corresponding extraosseous soft‐tissue mass. The lesion caused the medial maxillary wall to shift, restricting the left nasal airway and causing the nasal septum to deviate to the right and develop a little bony spur. The bulk made direct contact with and raised the left orbital floor superiorly. The orbital floor did not undergo periosteal erosion, orbital fat infiltration, or extraocular muscle involvement despite significant thinning and displacement. Bilateral inferior turbinate hypertrophy and a left concha bullosa were incidental findings (Figure 3).

**FIGURE 3:**
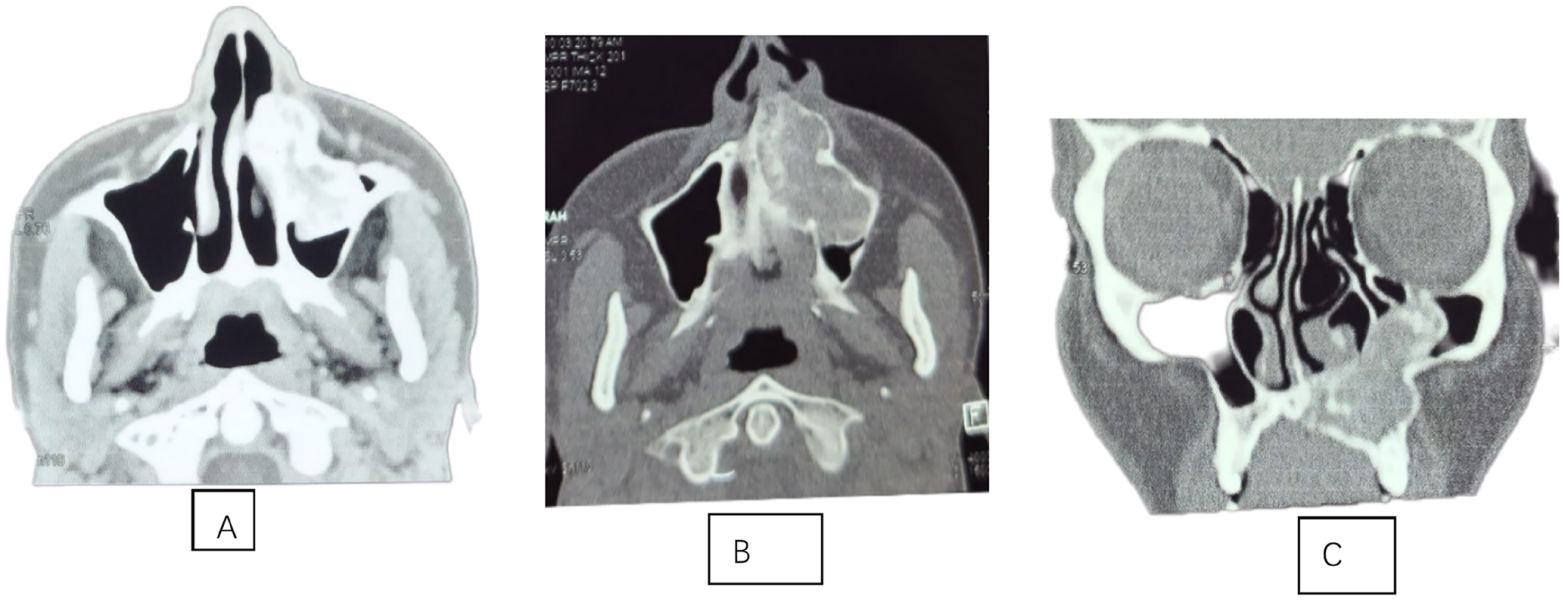
Preoperative computed tomography demonstrating a well‐defined mixed‐density mass occupying the left maxillary alveolus and maxillary sinus: (A) axial view showing expansion and thinning of the antral walls; (B) coronal view showing elevation of the orbital floor and deviation of the nasal septum; (C) sagittal view showing the anteroposterior extent of the lesion.

## 3 Methods (Differential Diagnosis, Investigations, and Treatment)

### 3.1 Differential Diagnosis

A slowly enlarging, painless, well‐defined expansile lesion was identified in the tooth‐bearing maxilla of a young adult, with clinical and radiographic features suggestive of a benign fibro‐osseous lesion or odontogenic tumor. Because of the well‐defined and corticated contact rather than the typical blending transition into adjacent bone, fibrous dysplasia was deemed implausible. Due to the lesion's noticeable growth and single mass‐like appearance, cemento‐osseous dysplasia was deemed inappropriate. Cementoblastoma, osteoblastoma, central giant cell granuloma, calcifying epithelial odontogenic tumor, and adenomatoid odontogenic tumor were further differential diagnoses. Low‐grade osteosarcoma and other sinonasal cancers were also taken into consideration due to the large sinus occupation and close closeness to the orbit. However, the prolonged indolent course, absence of pain or paraesthesia, preservation of cortical boundaries, lack of soft‐tissue infiltration, and absence of periosteal reaction strongly favored a benign process.

### 3.2 Treatment and Surgical Management

Three treatment options were taken into consideration: an endoscopic transnasal technique, conservative total enucleation, and a broad partial maxillectomy with reconstruction. The anterior maxillary alveolus and surrounding teeth would have to be sacrificed, which would result in significant reconstructive morbidity, even if partial maxillectomy would potentially reduce the likelihood of remaining tumor. Since the lesion started in the front maxillary alveolus rather than the sinonasal cavity, an endoscopic procedure was deemed inappropriate. After a thorough conversation with the patient, conservative in toto enucleation was chosen due to the clearly defined radiographic borders and apparent preservation of a surgical plane surrounding the lesion, with the knowledge that extended postoperative observation would be necessary.

Under general anesthesia, the lesion was enucleated using an intraoral vestibular technique and nasotracheal intubation. A thin, eggshell‐like cortical layer covering the tumor was removed to reveal its capsule after a full‐thickness mucoperiosteal flap was elevated. With blunt periosteal dissection, a distinct cleavage plane was found and circumferentially developed. In order to avoid piecemeal excision, the tumor was subsequently meticulously excised from the labial, antral, and superior aspects as a single solid mass measuring 7 × 6 × 2 cm (Figure 4). When the orbital floor was examined after the tumor was removed, it was discovered to be intact but noticeably thinned and displaced. No orbital rebuilding was necessary, and the periorbita remained intact. After a thorough examination, the remaining cavity was irrigated. Antral drainage was verified, and the sinus mucosa remained healthy.

**FIGURE 4:**
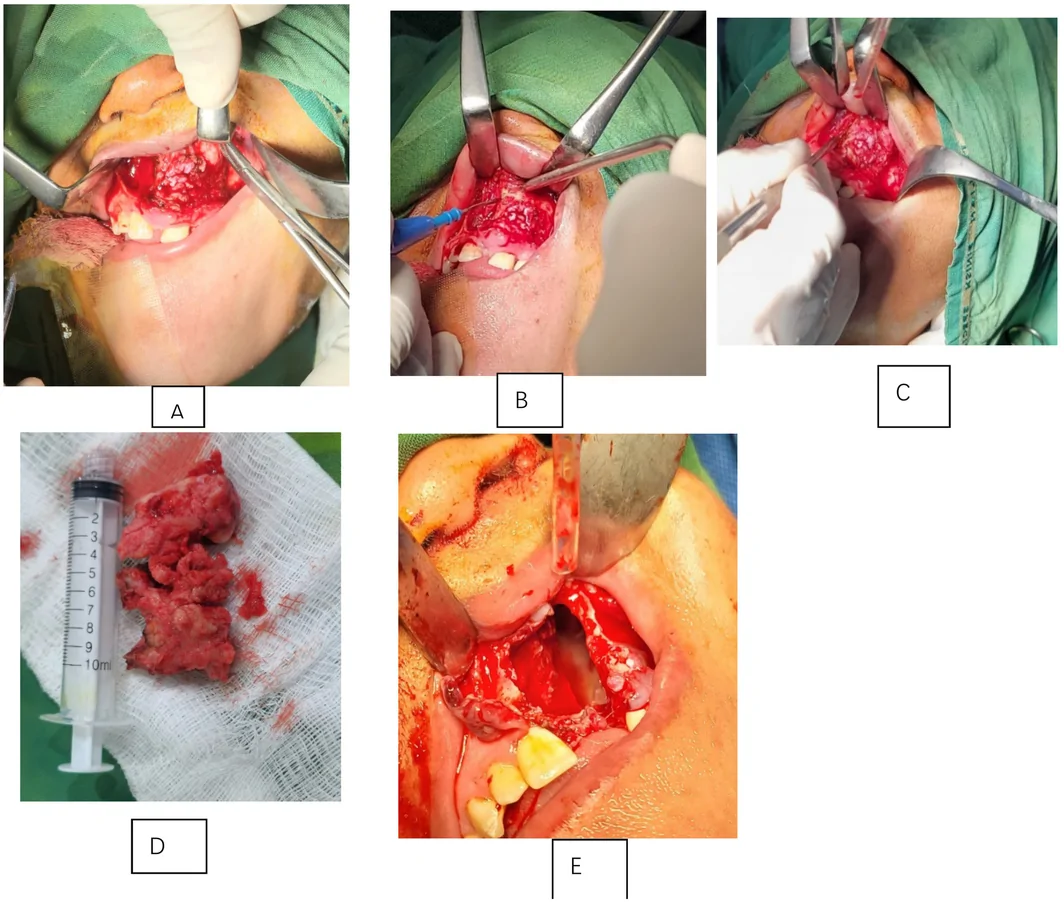
Intraoperative views: (A, B) reflection of the full‐thickness mucoperiosteal flap revealing the eggshell‐like cortical covering; (C) development of the extracapsular plane; (D) the enucleated specimen delivered as a single mass; (E) the residual bony cavity with the intact orbital floor.

The adjacent dentition was assessed according to the remaining osseous support. The left central incisor had lost its supporting bony housing and was therefore extracted. In contrast, sufficient labial and palatal bone remained around the lateral incisor to permit preservation, avoiding unnecessary sacrifice of the adjacent tooth. The flap was repositioned and closed primarily with resorbable sutures. The entire specimen was submitted for histopathological examination.

### 3.3 Histopathological Investigations

Histopathological examination demonstrated irregular trabeculae of woven and lamellar bone prominently rimmed by plump osteoblasts within a moderately to highly cellular fibrous connective tissue stroma composed of bland spindle‐shaped fibroblasts and collagen fibers. Focal cementum‐like spherules were also identified. No nuclear pleomorphism, atypical mitotic activity, permeative bone infiltration, or malignant features were observed. The well‐demarcated interface and prominent osteoblastic rimming supported the diagnosis of ossifying fibroma and favored it over fibrous dysplasia (Figure 5).

**FIGURE 5:**
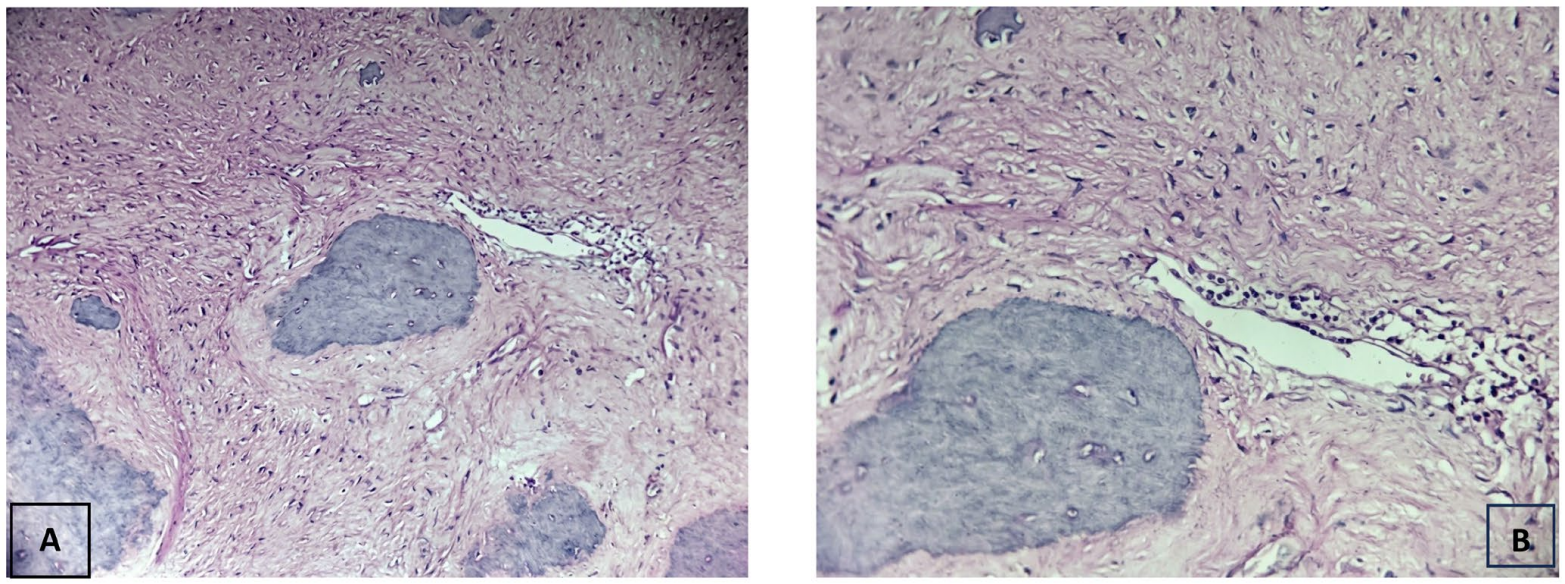
Photomicrographs of the surgical specimen (hematoxylin and eosin): (A) low‐power view showing mineralized trabeculae and cementum‐like deposits within a cellular fibrous stroma of spindle‐shaped cells. (B) High‐power view showing the mineralized tissue and the surrounding bland fibroblastic stroma without cytological atypia.

Special attention was given to the exclusion of juvenile ossifying fibroma JOF variant [2, 21]. Juvenile trabecular ossifying fibroma JTOF is characterized by a highly cellular stroma containing thin, anastomosing strands of immature cellular osteoid and woven bone. In the present lesion, the mineralized component consisted of trabeculae of both woven and mature lamellar bone and such immature osteoid strands were not observed. Juvenile psammomatiod ossicles fibroma (JPOF) is characterized by numerous small, concentrically laminated psammomatiod ossicles and predominantly involves the sinonasal and orbital bones. In contrast only focal cementum‐like spherules were identified here, without the dense, widespread psammomatiod ossicles typical of JPOF, and the lesion originated from the tooth‐bearing alveolus, the characteristic site of cemento‐ossifying fibroma [2]. Taken together with the patient's age 25 years and indolent 5‐year clinical course, these finding were consistent with conventional COF and did not support a diagnosis of a JOF variant. Integration of the clinical, radiological, and histopathological findings established a final diagnosis of giant central (cemento‐)ossifying fibroma of the left anterior maxilla with extensive maxillary sinus occupation and orbital floor displacement.

## 4 Conclusions and Results (Outcome and Follow‐Up)

### 4.1 Postoperative Course and Follow‐Up

The postoperative course was uneventful, with no significant hemorrhage, wound dehiscence, infection, oro‐antral communication, orbital complication, or infraorbital paraesthesia. Facial symmetry improved substantially, the upper lip returned to a normal resting position, and nasal airflow improved. Ocular position, extraocular movements, and visual acuity remained normal. The preserved lateral incisor remained functional, and prosthetic replacement of the extracted central incisor was subsequently completed.

The patient was evaluated at 1, 3, 6, and 12 months after enrolling in a structured clinical and radiological monitoring program. She had maintained face symmetry, adequate soft‐tissue healing, and no symptoms at the 1‐year evaluation. Proptosis, globe dystopia, diplopia, visual impairment, and infraorbital sensory deficit were not present. There was no clinical sign of persistent sinusitis or oro‐antral communication, and nasal breathing continued to improve. The preserved lateral incisor remained vital, firm, and functional.

A 12‐month radiographic evaluation showed increasing osseous regeneration of the surgical cavity, with the orbital floor preserved and maxillary sinus aeration restored. There was no evidence of a recurring or residual radiolucent or mixed‐density lesion (Figure 6). The patient was instructed to maintain yearly radiological and clinical monitoring.

**FIGURE 6:**
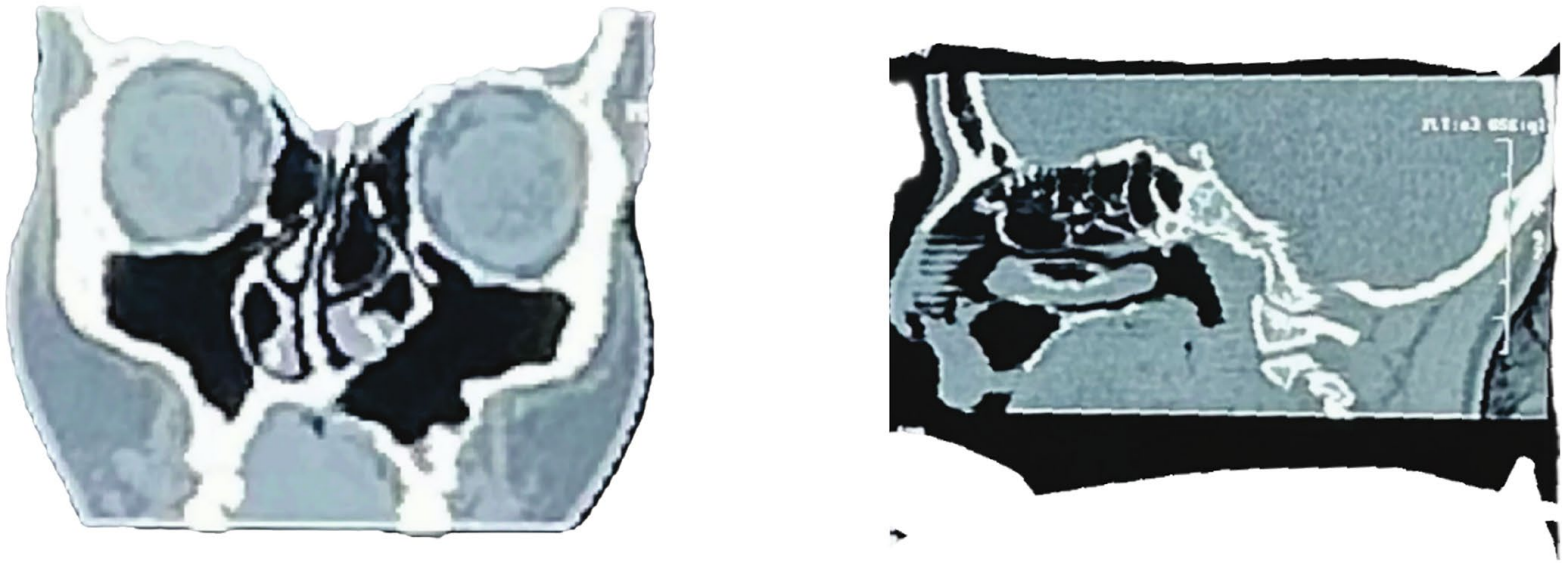
Postoperative follow‐up radiograph demonstrating progressive osseous fill of the surgical defect, restored maxillary sinus aeration and an intact orbital floor, with no evidence of recurrence.

## 5 Discussion

The unique combination of anterior maxillary position, substantial maxillary sinus occupation, orbital floor displacement, and huge size (7 × 6 × 2 cm) makes this case notable rather than diagnostic doubt. The lesion produced facial asymmetry and tooth displacement without pain, paresthesia, diplopia, or visual impairment despite this widespread anatomical involvement. Slow‐growing, expansile lesions within the pneumatized maxilla, where progressive growth may proceed with displacement rather than invasion of neighboring structures, are characterized by this apparent disparity between tumor size and symptom burden [13, 15].

The clinical, radiological, and histopathological findings were consistent with conventional COF. COF was preferred above fibrous dysplasia and cemento‐osseous dysplasia due to its expansile behavior, mixed internal density, and finely defined and corticated interface [3, 8, 16]. The diagnosis was further confirmed histologically by the presence of mineralized trabeculae with noticeable osteoblastic rimming inside a cellular fibrous stroma. Crucially, the lack of soft‐tissue invasion, unusual mitoses, and permeative bone infiltration provided compelling evidence against cancer.

Exclusion of JOF variants was particularly important, because these variants may exhibit more aggressive behavior and show higher recurrence rates after conservative surgery [2, 8, 19]. JOF typically affects children and adolescents and often grows rapidly, although it may occasionally occur in adults [19]. In the present case, the adult age at presentation, slow 5‐year progression, and origin from the tooth‐bearing alveolus favored conventional COF. Histologically, the lesion contained trabeculae of both woven and lamellar bone lined by plump osteoblasts and lacked both the thin strands of immature cellular osteoid characteristic of JTOF and the dense, widespread psammomatiod ossicles characteristic of JPOF [2, 19, 21].

The patient's age and gender fit the typical COF epidemiology profile. The second to fourth decades of life are when COF is most commonly diagnosed, and it primarily affects women [10, 11, 18]. The current situation, however, deviates significantly from the typical anatomical distribution. Most described lesions occur in the mandible, while anterior maxillary involvement is quite rare [9, 10, 11]. An even less common appearance is extension into the maxillary sinus with elevation of the orbital floor [12, 13, 14, 15]. Therefore, rather than the patient's demographics, the primary peculiarity of this case is its location and scope.

The effective conservative treatment of an exceptionally large lesion was the most clinically significant discovery. Tumor size, anatomical position, recurrence, and the viability of total removal all play a role in the surgical treatment options for COF, which range from enucleation and curettage to block or segmental resection [11, 18, 19]. In this instance, the decision to forego a maxillectomy was based not only on the benign diagnosis but also on the existence of a clearly visible cleavage plane and a well‐defined tumor capsule. As a result, the maxillary sinus walls and orbital floor could not be compromised in the mobilization and intact removal of the tumor. While the central incisor needed to be extracted because its bony support had entirely disappeared, the lateral incisor was also retained because sufficient supporting bone was still there.

This observation is clinically significant because tumor size alone should not necessarily dictate radical surgery in conventional COF. These lesions are clearly defined and may be isolated from surrounding tissues rather easily, according to the WHO classification [1]. This idea is extended in our example to a massive maxillary lesion with sinonasal and orbital extension, showing that significant radiographic involvement does not always indicate invasive biological behavior or need maxillectomy. Rather, the main focus of surgical planning should be the lesion's link to important structures and the viability of total extracapsular excision.

Nevertheless, this favorable outcome should be regarded as a case‐specific observation rather than as a general treatment guideline. Conservative enucleation was achievable in this patient only because of a well‐defined, intact capsule and a distinct dissection plane. In lesions that are poorly marginated, show aggressive or infiltrative growth, or lack a clear plane of separation from adjacent vital structures, radical surgical resection is generally indicated; surgical decision‐making must therefore be tailored to each case.

The postoperative findings further support this approach. At 12 months, the patient was still asymptomatic, facial symmetry was preserved, the preserved lateral incisor was still functional and vital, and CT showed progressive osseous fill, sinus aeration restoration, and an intact orbital floor with no radiographic signs of recurrent or residual disease. Although these results are promising, they should not be taken as proof that gigantic COF should always receive conservative treatment. Across several datasets, published recurrence rates range from roughly 6.7% to 28.6% [8, 11, 12, 20]. Variations in the histological subtype, anatomical location, extent of excision, patient age, and length of follow‐up are probably responsible for this variation.

This case also demonstrates the need for cautious interpretation of recurrence rates. Juvenile trabecular and juvenile psammomatoid variations, which may exhibit more aggressive behavior and greater recurrence rates after conservative treatment, should be differentiated from conventional COF [2, 19]. Similarly, because of their close proximity to the orbit, skull base, and nasal cavity, diseases affecting the sinonasal tract may provide more technical difficulties [12, 13, 14]. The presence of a continuous capsular plane and the lack of infiltrative behavior in our patient allowed for total removal, which probably contributed to the favorable early outcome.

The main limitation is the 12‐month follow‐up, which cannot establish long‐term tumor control because recurrence may occur later [11, 12]. A single case also cannot compare conservative enucleation with radical resection. Nevertheless, this case suggests that tumor size and sinonasal–orbital extension alone may not require radical surgery in conventional COF. For well‐circumscribed lesions with an intact capsule and clear cleavage plane, conservative enucleation may provide satisfactory control while preserving adjacent structures and reducing morbidity. Longer follow‐up and additional cases are needed to confirm the durability and generalizability of this approach.

## 6 Conclusions

This case reports a giant central ossifying fibroma of the anterior maxilla extending into the maxillary sinus and displacing the orbital floor. Complete conservative enucleation was achieved through an intact capsule and clear cleavage plane, preserving the orbital floor and lateral incisor. Histopathological examination supported the diagnosis of COF and did not support a juvenile ossifying fibroma variant. At 1‐year follow‐up, progressive bone regeneration occurred without recurrence. Although giant size and sinonasal–orbital extension do not automatically necessitate radical resection when a clear cleavage plane exists, conservative management of such extensive lesions remains a case‐specific observation that requires careful individualized patient selection and long‐term surveillance.

## Author Contributions

**Ahmed Alashwal:** writing – original draft, funding acquisition, investigation, conceptualization, writing – review and editing, visualization, validation, methodology, project administration, formal analysis, software, resources, supervision, data curation. **Naibah Rajeh:** resources, supervision, formal analysis, visualization, writing – review and editing, writing – original draft, methodology. **Arham M. Alkherbash:** conceptualization, visualization, writing – review and editing, writing – original draft, formal analysis. **Ahmed Madfa:** writing – review and editing, writing – original draft, methodology, formal analysis, data curation.

## Funding

The authors have nothing to report.

## Disclosure

Standards of Reporting: CARE guideline has been followed [22].

## Ethics Statement

The authors have nothing to report.

## Consent

To publish this case report, which includes a description of this case, medical and dental information, and photographs, the patient supplied written informed consent for the treatment procedures and for publication.

## Conflicts of Interest

The authors declare no conflicts of interest.

## Data Availability

The data that support the findings of this study are available on request from the corresponding author. The data are not publicly available due to privacy or ethical restrictions.
